# Screening and application of nutritional support in elderly hospitalized patients of a tertiary care hospital in China

**DOI:** 10.1371/journal.pone.0213076

**Published:** 2019-03-08

**Authors:** Ying-Min Lin, Min Wang, Nuan-Xin Sun, Yan-Yan Liu, Teng-Fei Yin, Chen Chen

**Affiliations:** 1 Department of General Practice, Qilu Hospital of Shandong University, Jinan, Shandong Province, China; 2 Department of Gastroenterology, Geriatrics, Qilu Hospital of Shandong University, Jinan, Shandong Province, China; 3 Jiangxi medical school, Nanchang University, Queen Mary University of London, Nanchang, Jiangxi Province, China; 4 Department of Gastroenterology, Chengyang People’s Hospital, Qingdao, Shandong Province, China; 5 Chen Chen, Donga County people’s Hospital, Liaocheng, Shandong Province, China; Medical University Graz, AUSTRIA

## Abstract

**Background:**

Malnutrition is very common in elderly patients admitted to the hospital. The aim of our study is to assess the nutritional status of elderly patients and the use of nutritional support in a tertiary care hospital in China and to analyze the impacts of nutritional status and nutritional support on clinical outcomes.

**Methods:**

Statistical analysis was performed on a sample of 745 elderly patients in the geriatric medicine department of Qilu Hospital of Shandong University from March 2012 to March 2015. The Nutrition Risk Screening 2002 (NRS 2002) and Mini Nutritional Assessment-short forms (MNA-SF) were utilized for the nutritional risk screening at admission. Personal information, anthropometric measurements, laboratory tests, nutritional support and clinical outcomes were recorded. Comparisons were carried out to analyze impacts on clinical outcomes and prognosis based on incidence rate of nutritional risk, nutritional support rate, and different methods of support.

**Results:**

NRS 2002 and MNA-SF were utilized to screen for nutritional risk at admission. The results of this screening were 39.81% and 44.10%, respectively. Based on the results of the MNA-SF, 33.38% of elderly patients were at risk of malnutrition and 5.5% were malnourished. The incidence of nutritional risk in the departments of Gastroenterology, Hematology, and Respiratory were 51.72%, 46.88%, 43.33%, respectively, higher than in other departments. Patients with nutritional risk were more likely to have a longer hospital stay compared to those without (*P* < 0.05). The nutritional support rate of patients overall was 16.49%, and the ratio of Parenteral nutrition (PN):Enteral nutrition (EN) was 5.13:1. Patients at nutritional risk had an in-hospital support rate of 29.63% and 28.57%, respectively, identified via screening by NRS 2002 and MNA-SF. Nutritional support rate of patients without nutritional risk was 7.8%(35/449) and 6.96%(29/417), respectively. Patients in the departments of Gastroenterology and Hematology had higher rates of nutritional support than patients in other departments. In addition, results showed that in patients with nutritional risk and malnutrition, nutritional support decreased the length of hospital stay (*P*<0.05). The patients that received nutritional support also had a lower incidence of infectious complications than the patients without nutritional support (NRS 2002 was 6.82%:18.18% and MNA-SF was 9.57%:20.23%)(*P*<0.05).

**Conclusions:**

Undernourishment and nutritional risk in elderly patients at hospital admission is a common occurrence. In the current study, the nutritional risk rate in the Gastroenterology department was higher than in other departments. Patients with normal nutritional status were still receiving nutritional support. Overall, there is a need to better apply nutritional support in the clinical treatment of elderly patients. In elderly patients with nutritional risk and malnutrition, nutritional support reduced the length of hospital stay and the incidence of infectious complications.

## Introduction

Since the practice of parenteral and enteral nutritional support was applied in clinical treatment, the application of nutritional support has experienced rapid development. The theories and methods behind nutritional support are constantly evolving. For patients who need nutritional support, choice of the appropriate method can enhance the recovery of patients and significantly improve clinical results and prognosis [1–3]. Due to a higher nutritional risk rate and functional degeneration, patients with multiple conditions are more common among elderly patients, who are more susceptible to disease and malnutrition. This makes these patients a target for nutritional support [4, 5]. Studies on the effects of nutritional support on clinical results have mainly focused on the nutritional status of patients, the tolerance of clinical treatments, rate of complications, hospital length of stay (HLOS), readmission rate, mortality rate, and the cost of care [6, 7]. Results of the studies vary, and there is disagreement over the timing and methods for nutritional support [8, 9].

This study used the NRS 2002 and MNA-SF to conduct screening on 746 elderly hospitalized patients, evaluating the nutritional status of the patients and studying the effects of nutritional support on clinical results.

## Methods

### 2.1 Patients

The study was a prospective observational study in the geriatric medicine department of the Qilu hospital of Shandong University. Recruitment was carried out among patients admitted consecutively to the wards from March 2012 to the end of March 2015.

**Inclusion criteria** included: Age ≥ 65; scheduled to stay in the hospital for at least 3 days; no plan to conduct surgery after hospitalization; consent to participate in the study; must have undergone nutritional screening within 24h after hospitalization.

**Exclusion criteria** included: excluded for nutritional risk screening; having surgery during hospitalization; patient with critical illness, acute disease or infection, needing treatment prior to nutritional assessment at the time of admission; HLOS < 3d; dropped out during the study; incomplete data (presented as follows).

### 2.2 Data collection

Patients newly admitted were asked whether they would like to participate in this investigation. Personal information (age, sex, ethnicity, department, date of admission, primary diagnosis, co-existing comorbidities) were collected. After their agreement, nutritional screening was performed by the same researchers after standard training. Patients’ weight and height from admission to discharge were measured also by the researchers with the same standard scale: before meals in the morning, with shoes off and wearing a hospital gown. The height of the patient was measured to the nearest 0.5 cm, and body weight to the nearest 0.5kg. The following laboratory tests were carried out using standard methods within 72h after hospitalization: hemoglobin (Hb), total lymphocyte count (TLC), and albumin (Alb). The patients’ nutritional supports being delivered and clinical results were also collected from their medical records. The data-collection should be performed until the time of patients’ discharge. Data were evaluated independently by two individuals before entering the information into the electronic database.

### 2.3 Nutritional screening methods

NRS 2002 and MNA-SF were used to evaluate patient nutritional status within 48h of hospitalization. NRS 2002 consists of three sections: impaired nutritional status, severity of disease and age. It contains a total of 7 points. Impaired nutritional status is scored from 0~3 according to changes of Body Mass Index (BMI), weight loss and food intake. Severity of disease is scored 0~3 according to different kinds of disease. If age ≥70 years: add 1 to the total score. Patients with a score ≥ 3 have nutritional risk and in need of nutritional support with weekly review. Patients with a score < 3 do not have nutritional risk and need no intervention. However, reviews should be conducted after one week. [10]

MNA-SF consists of six sections: appetite or eating problem, recent weight loss, mobility impairment, acute illness/ stress, dementia or depression and BMI. It contains a total of 14 total points; a score of 12–14 is within the normal range, 8–11 indicates risk of malnutrition, and ≤ 7 indicates malnutrition. [11] The standard measurement for malnutrition is a BMI of < 18.5kg/m^2^. [12] In patients without a BMI reading, the standard for malnutrition can be based on an Alb level < 30g/L.

### 2.4 Nutritional support analysis

This study documented various aspects of nutritional support which included the specific method and type of medication, the starting and finishing time, and the daily nutrients and energy provided. Parenteral nutrition supplies daily nutritional requirements from the peripheral vein or a central venous catheter, with non-protein calories ≥ 10kcal/kg/d for more than 3 days. Enteral nutrition includes oral nutritional supplements (ONS) as well as tube feeding via nasogastric, nasal-enteral, or percutaneous tube, with non-protein calories ≥ 10kcal/kg/d for more than 3 days.

### 2.5 Clinical results

The clinical results included infectious complications, non-infectious complications, nutritional support-related complications, HLOS, and the total cost of hospitalization.

Infectious complications include localized or systemic conditions resulting from an adverse reaction to the presence of an infectious agent(s) or its toxin(s); there must be no evidence that the infection was present or incubating at the time of admission to the hospital (pneumonia, urinary tract infection, intraperitoneal infection, catheter-related infection, other infection). [13]

Non-infectious complications included new diseases and conditions after hospitalization (anemia, myocardial infarction, organ failure, etc.), caused by primary disease and weakness, excluding infection induction.

Nutritional support-related complications include gastrointestinal complications (nausea, vomiting, diarrhea, bloating, constipation), metabolic complications (hyperglycemia, hyperlipidemia, dehydration, liquid retention, hypophosphatemia, nutrition-associated liver disease, etc.), infectious complications (aspiration pneumonia, central venous catheter related infection, etc.), mechanical complications (gastric perforation, ulcer, intestinal obstruction and nutrient tube blockage, central venous catheter related injuries and embolism, etc.) caused by nutritional support.

### 2.6 Ethics

The present study followed the ethical guideline of the Declaration of Helsinki and was approved by the Ethical Committee of Chinese host hospital (Beijing hospital, code center number 01110005) (2012BJYYEC-002-A and 2014BJYYEC-022-02). All the participants were volunteers and signed an informed consent before the audit. Hospitals and units were labeled with a numeric code, and participants were recorded with the initials of their name to protect patients’ privacy.

### 2.7 Statistical analysis

Statistical analyses were performed with SPSS 20.0 (SPSS Inc., Chicago, IL, USA). Quantitative data were expressed as mean ± standard deviation, and qualitative data were expressed as proportions. Differences in Quantitative data were analyzed using analysis of variance. Differences in qualitative data were measured by chi-square test or Fisher’s exact test. Multivariate statistical analysis of the factors affecting clinical outcomes were measured by Logistic multiple regression analysis. *P* < 0.05 was considered statistically significant.

## Results

### 3.1 Basic material analysis

There were 746 elderly patients included in this study with an average age of 77.29 ± 6.97 and BMI of 23.67 ± 3.49 kg/m^2^. The sample included 491 male patients with an average age of 77.62 ± 7.12 and a BMI of 23.87 ± 3.36 kg/m^2^, and 225 female patients with an average age of 76.66 ± 6.66 and a BMI of 23.67 ± 3.73 kg/m^2^ (see Table 1).

**Table 1:** Baseline characteristics of the patients. N = 746.

Characteristics	
**Age**	77.29±6.97
65~70	156(20.91%)
71~80	308(41.29%)
81~90	269(36.06%)
Over than 90	13(1.74%)
**Sex**	
Male	491(65.82%)
Female	255(34.18%)
**BMI** (kg/m^2^)	23.87±3.49
<18.5	41(5.50%)
18.5≤BMI<24.0	342(45.84%)
24.0≤BMI<28.0	273(36.60%)
≥28.0	90(12.06%)
**Department**	
Neurology Department	123(16.49%)
Hematology Department	64(8.58%)
Pneumology Department	90(12.06%)
Gastroenterology Department	174(23.32%)
Endocrinology Department	174(23.32%)
Cardiology Department	229(30.70%)

### 3.2 Analysis of nutritional risk and rate of malnutrition

**Fig 1:**
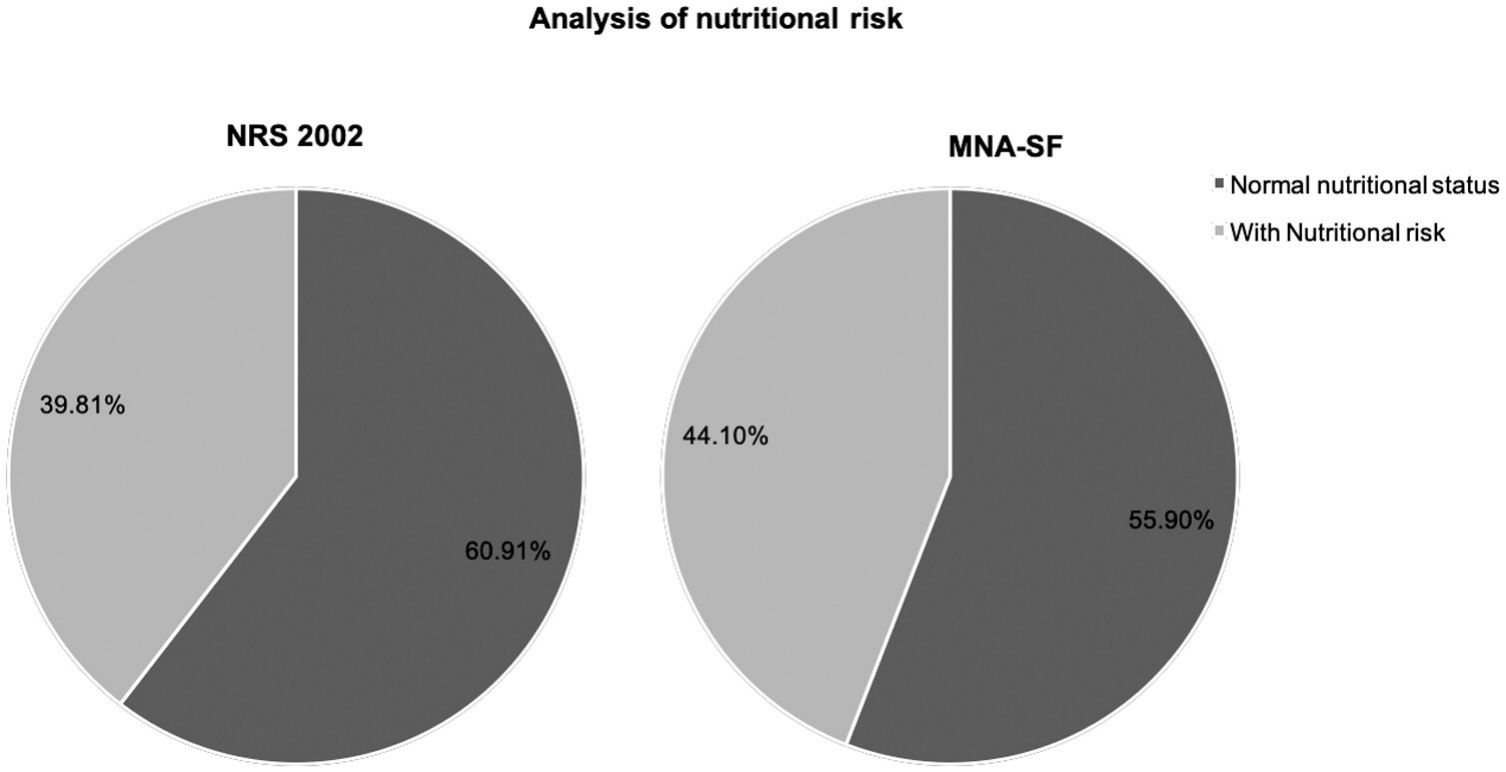
Analysis of nutritional risk using NRS 2002 and MNA-SF.

Of the 746 patients, there were 41 cases (5.5%) of malnutrition (BMI < 18.5kg/m^2^) and nutritional risk was 39.61% (297/746) (using the NRS 2002 results). Using the MNA-SF to evaluate the nutritional status of patients, the study found that the ratio for patients with normal nutritional status was 55.9% (417/746), the malnutrition rate was 33.38% (249/746), the occurrence rate of malnutrition was 10.72%, and the overall nutritional risk was 44.1% (329/746) (see Fig 1).

**Fig 2:**
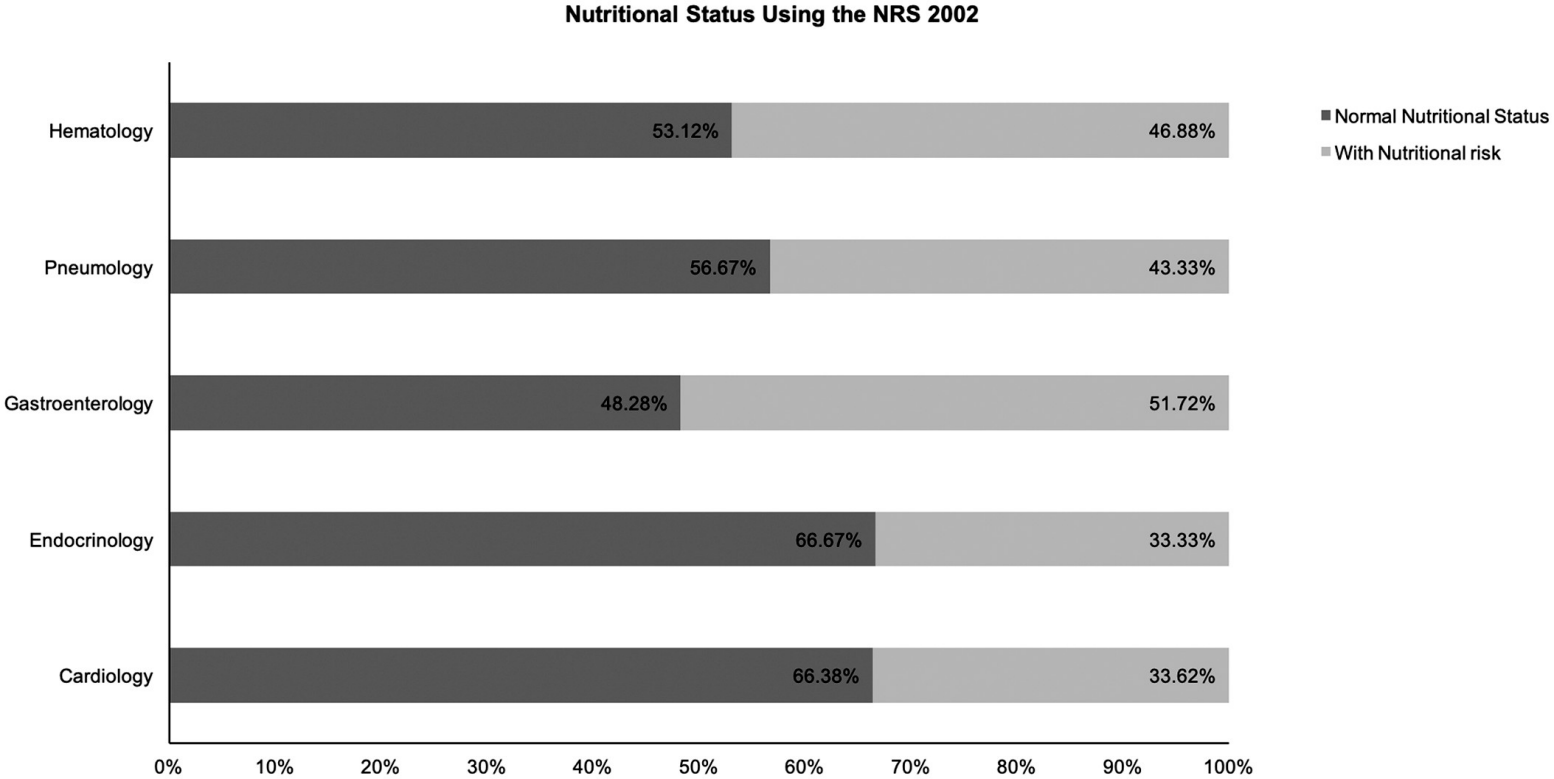
Nutritional status using the NRS 2002.

**Fig 3:**
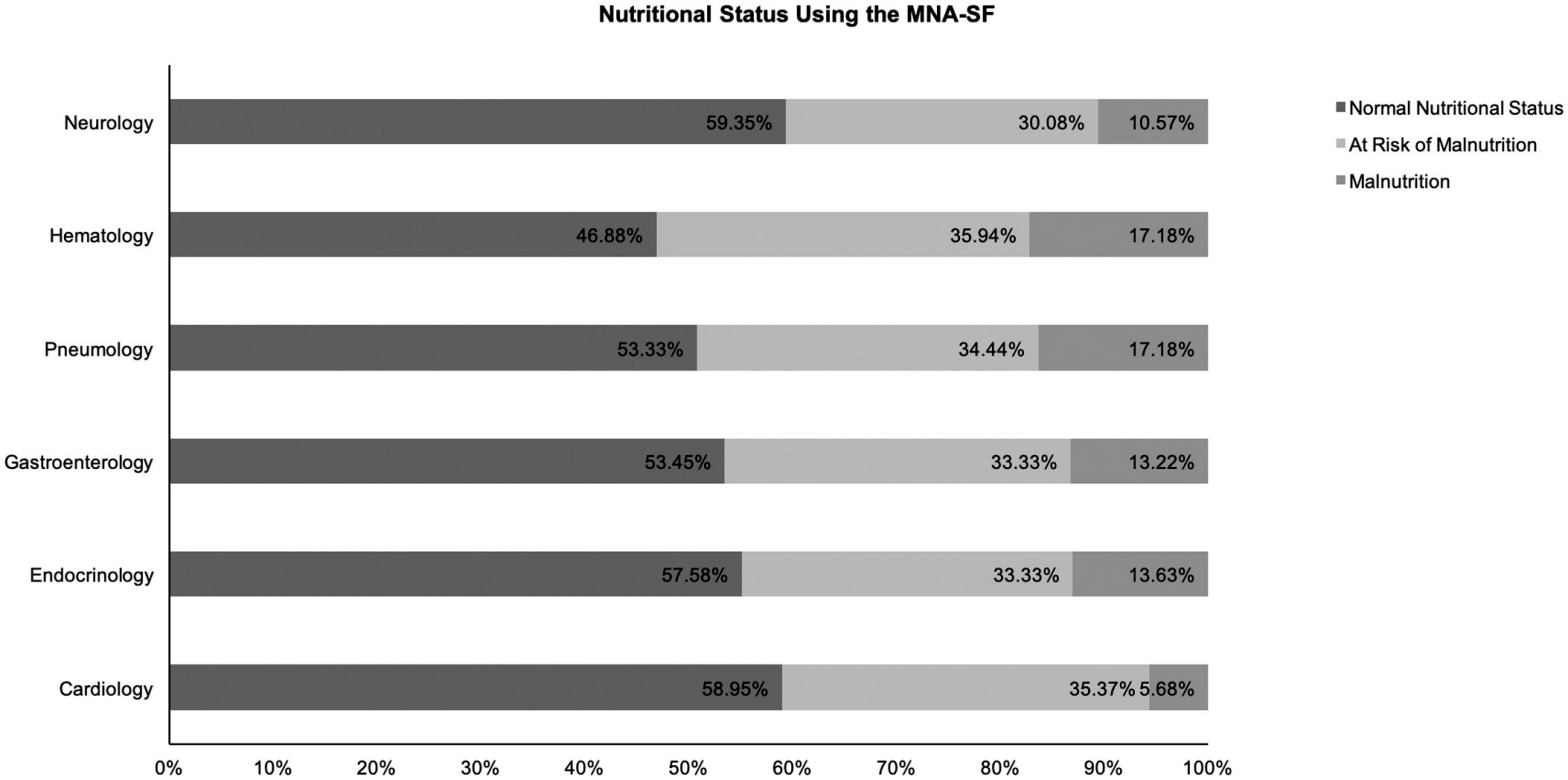
Nutritional status using the MNA-SF.

Patients’ nutritional characteristics according to the NRS 2002 and MNA-SF results are shown in detail in Table 2. Using the NRS 2002 results, we analyzed the nutritional risk rate of different departments and found statistically significant differences. Hospitalized patients in the Gastroenterology Department had the highest nutritional risk rate, which was greater than 50%. The second highest rate was in the Hematology Department, which was 46.88%. The Neurology, Endocrinology, and Cardiology Departments all had lower rates (see Fig 2). However, using the MNA-SF results, we didn’t find statistically significant differences when we analyzed the nutritional risk rate of different departments (see Fig 3).

**Table 2:** Analysis of nutritional risk and rate of malnutrition.

	NRS 2002		MNA-SF		
Nutritional status	Normal nutritional status, n = 449	With Nutritional risk, n = 297	Normal nutritional status, n = 417	At risk of malnutrition, n = 249	Malnutrition, n = 80
Age*	76.31±6.92	78.78±6.80	76.16±6.71	78.31±6.80	80.06±7.67
**Sex**					
Male	299 (60.90%)	192 (39.10%)	275(56.01%)	157(31.98%)	59(12.01%)
Female	150 (58.82%)	105 (41.18%)	142(55.69%)	92(36.08%)	21(8.23%)
**BMI** (kg/m^2^) *	25.22±2.83	21.83±3.40	25.36±2.82	22.83±3.11	19.36±2.61
**Department**					
Neurology Department	84 (68.29%)	39 (31.71%)	73(59.35%)	37(30.08%)	13(10.57%)
Hematology Department	34 (53.12%)	30 (46.88%)	30(46.88%)	23(35.94%)	11(17.18%)
Pneumology Department	84 (56.67%)	39 (43.33%)	48(53.33%)	31(34.44%)	11(17.18%)
Gastroenterology Department	84 (48.28%)	90 (51.72%)	93(53.45%)	58(33.33%)	23(13.22%)
Endocrinology Department	44 (66.67%)	22 (33.33%)	38(57.58%)	58(33.33%)	9(13.63%)
Cardiology Department	152 (66.38%)	77 (33.62%)	135(58.95%)	81(35.37%)	13(5.68%)

*Statistically significant at *P* < 0.05.

### 3.3 Nutritional risk rate and the HLOS

According to results from the NRS 2002, the average HLOS in patients with nutritional risk was 13.76 ± 7.51 days, which was significantly longer than that without nutritional risk (11.51±6.36 days), (*P* < 0.05). According to the MNA-SF results, the average HLOS for the normal nutrition group, the nutritional risk group, and the malnutrition group were 11.57 ± 6.50, 13.15 ± 7.35, and 14.42 ± 7.08 days, respectively (*P* < 0.05). Patients in the normal nutrition group had the shortest HLOS (*P* < 0.05). According to MNA-SF results, the study divided the patients into two groups, the MNA-SF ≥ 12(normal nutrition group) and the MNA-SF < 12 (nutritional risk group); the average HLOS for the two groups was 11.57 ± 6.50 and 13.46 ± 7.30 days, respectively (*P* < 0.05).

### 3.4 Analysis of nutritional support

**Fig 4:**
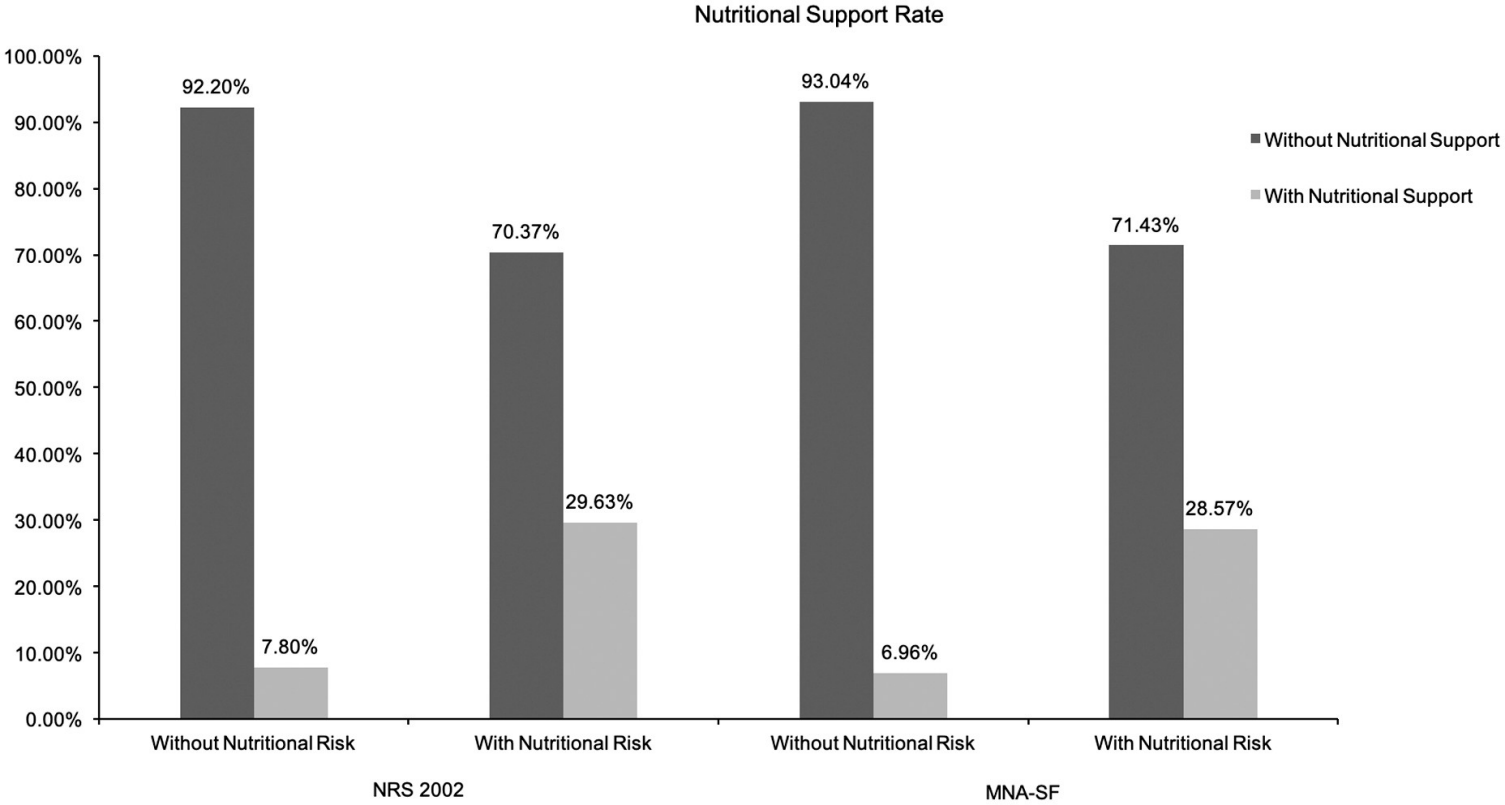
Nutritional support rate.

Of the 746 cases, 123 patients (16.49%) received nutritional support which included 82 PN, 16 EN, and 25 combined application. The ratio between PN and EN was 5.13:1. At NRS 2002 screening, the nutritional support rate for patients with nutritional risk was 29.63% (88/297). At MNA-SF evaluation, the rate was 28.57% (94/329). These details are shown in Fig 4. For the 41 cases with malnutrition (BMI < 18.5kg/m^2^), only 16 (39.02%) cases received nutritional support. Conversely, some patients with normal nutrition received additional nutritional support. At NRS 2002 screening, the nutritional support rate for patients without nutritional risk was 7.8%(35/449). At MNA-SF evaluation, the rate was 6.96%(29/417). They all had no indications for nutritional support.

By analyzing nutritional support in the nutritional risk group by department, we determined that patients in the Gastroenterology Department received the highest rate of nutritional support. Results are presented in detail in Table 3.

**Table 3:** Analysis of patients with nutritional risk.

	NRS 2002		MNA-SF	
Nutritional support	With nutritional support, n = 209	Without nutritional support, n = 88	With nutritional support, n = 235	Without nutritional support, n = 94
Age*	78.00±6.54	80.63±7.07	78.14±6.82	80.24±7.41
**Sex**				
Male	126 (65.63%)	66 (34.38%)	143 (66.20%)	73 (33.80%)
Female	83 (79.05%)	22 (20.95%)	92 (81.42%)	21 (18.58%)
**BMI** (kg/m^2^) *	22.12±3.40	21.14±3.32	22.31±3.34	21.18±3.22
**Department**				
Neurology Department	26 (66.67%)	13 (33.33%)	35 (70.00%)	15 (30.00%)
Hematology Department	24 (80.00%)	6 (20.00%)	25 (73.53%)	9 (26.47%)
Pneumology Department	30 (76.92%)	9 (23.08%)	32 (76.19%)	10 (23.81%)
Gastroenterology Department	48 (53.33%)	42 (46.67%)	39 (48.15%)	42 (51.85%)
Endocrinology Department	18 (81.82%)	4 (18.18%)	24 (85.71%)	4 (14.29%)
Cardiology Department	63 (81.82%)	14 (18.18%)	80 (85.11%)	14 (14.89%)

*Statistically significant at *P* < 0.05.

### 3.5 Effect of nutritional support on HLOS

Based on the NRS 2002 results, of the 297 cases with nutritional risk, 11 cases were excluded due to a HLOS that was prolonged for non-clinical reasons. For the remaining 286 cases, patients with nutritional support stayed in the hospital for an average of 12.22 ± 5.48 days, which was shorter than the patients without nutritional support (14.35 ± 6.19 days, t = -2.839, *P*<0.05). MNA-SF screening in patients with nutritional risk (after excluding the 11 cases) revealed that the HLOS of patients with nutritional support was (11.94 ± 5.18 days, which was shorter than patients without nutritional support (14.41 ± 6.44 days, t = -3.529, *P*<0.05).

### 3.6 Effect of nutritional support on the incidence of complications

**Fig 5:**
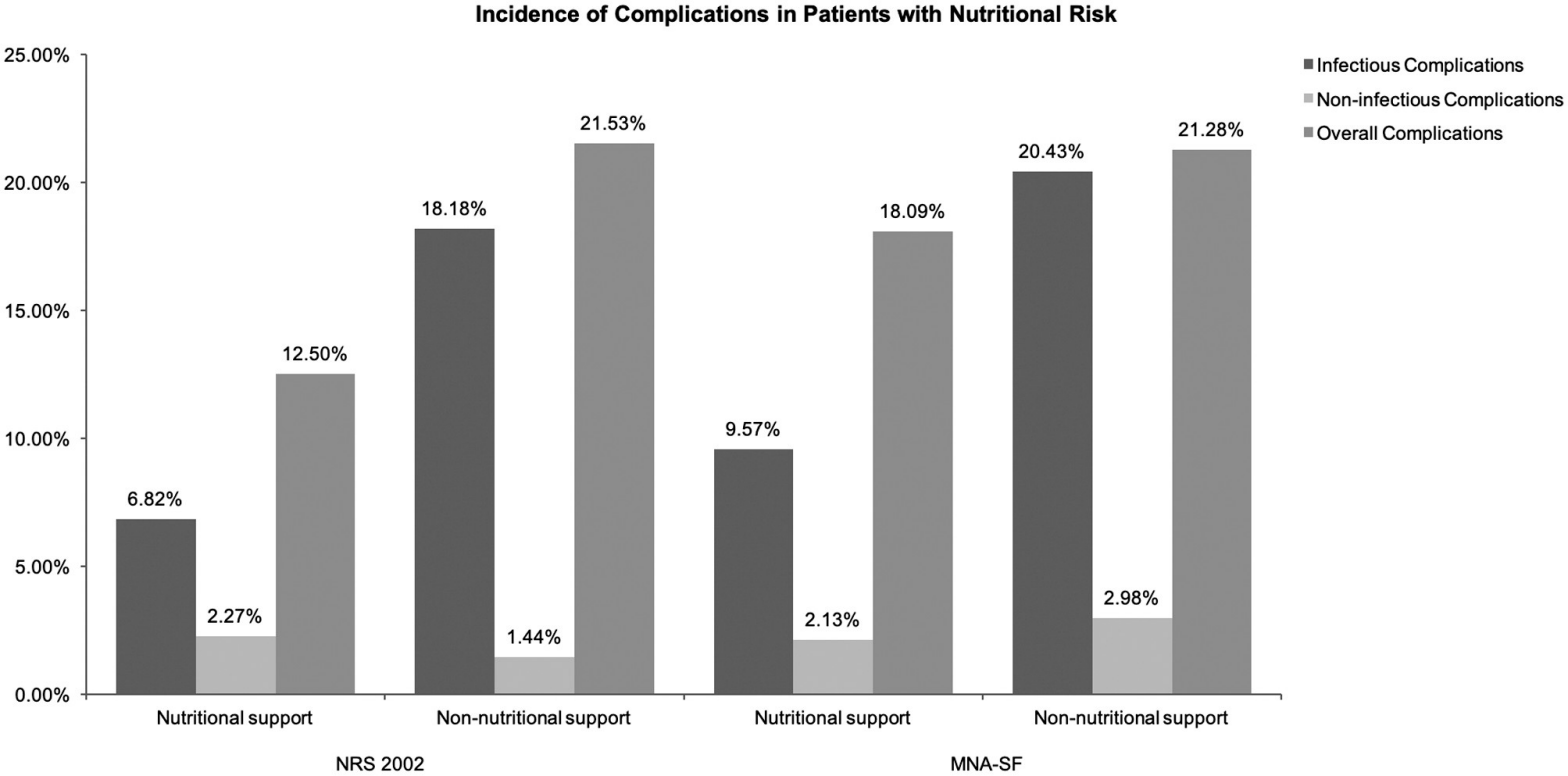
Incidence of complication in patients with nutritional risk.

The relationship between nutritional support and the incidence of complications was shown in Fig 5. At NRS 2002 screening, of the 297 patients with nutritional risk, complications occurred in 56 cases (18.86%), the incidence of infectious complications was 14.81% (44/297), the incidence of non-infectious complications was 1.68% (5/297), and the incidence of complications related to nutritional support was 5.05% (15/297). The details of the complications in the two groups are shown in Table 4. The nutritional support group had a lower incidence of infectious complications than the non-nutritional support group, (*P* < 0.05). Regarding the incidence of non-infectious complications and overall complications, the difference between the two groups was not statistically significant (*P* > 0.05).

**Table 4:** Incidence of complications in patients with nutritional risk (NRS 2002).

	Infectious complications	Non-infectious complications	Overall complications
**Nutritional support group, n = 88**	6 (6.82%) *	2 (2.27%)	11 (12.50%)
**Non-nutritional support group, n = 209**	38 (18.18%)	3 (1.44%)	45 (21.53%)

*Statistically significant at *P* < 0.05.

At MNA-SF screening, of the 329 patients with nutritional risk, the overall incidence of complications was 20.36% (67/329), in which the incidence of infectious complications was 17.33% (57/329) and the incidence of non-infectious complications was 2.74% (9/329). The nutritional support group had a lower incidence of infectious complications than the non-nutritional support group (*P*<0.05). Details regarding complications in the nutritional support group and the non-nutritional support group (MNA-SF) are shown in Table 5.

**Table 5:** Incidence of complications in patients with nutritional risk (MNA-SF).

	Infectious complications	Non-infectious complications	Overall complications
**Nutritional support group, n = 94**	9 (9.57%) *	2 (2.13%)	17 (18.09%)
**Non-nutritional support group, n = 235**	48 (20.43%)	7 (2.98%)	50 (21.28%)

*Statistically significant at *P* < 0.05.

Next, we ran a multivariate regression analysis on the factors that affect infectious complications (age, sex, BMI, nutritional support status, rating of illness degree, nutritional status rating, and HLOS). The results showed that the rating for illness, nutritional status rating, and HLOS were significantly related to the incidence of complications (*P* < 0.05, Table 6).

**Table 6:** Multivariate regression analysis of the factors affecting infectious complications.

	RR	SE	Wald value	*P* value	OR	95%CI
**Age**	0.01	0.03	0.08	0.78	1.01	0.94~1.05
**Sex**(male vs. female)	-0.14	0.36	0.14	0.71	0.87	0.57~2.30
**BMI**	0.07	0.06	1.24	0.27	1.07	0.83~1.05
**HLOS**	0.05	0.02	6.45	0.01*	1.05	1.01~1.09
**Rating for illness degress**	0.59	0.21	7.74	0.01*	2.08	1.07~4.85
**Nutritional status rating**	0.74	0.43	3.90	0.04*	1.15	1.04~1.71
**Nutritional support**(with vs. without)	-1.17	0.48	5.95	0.02*	0.31	0.12~0.80

*Statistically significant at P < 0.05.

## Discussion

Our results suggest that undernourishment and nutritional risk in elderly at hospital admission is a common occurrence. Patients with nutritional risk are more likely to have a longer hospital stay. Nutritional support decreases the length of hospital stay and reduces the number of patients with infectious complications.

There have been a large number of studies analyzing nutritional risk in elderly hospitalized patients. A recent review summarized multiple large-sample studies and showed that 22%–28% of elderly patients had nutritional risk, and 23%-60% of elderly patients were malnourished [4, 14, 15]. During the time frame of 2012.3 to 2015.3, using the methods of MNA-SF and NRS-2002, CSPEN conducted nutritional screening and evaluation of elderly hospitalized patients at 30 Class III Grade I hospitals in major Chinese cities. The two methods showed that more than 40% of patients had nutritional risk, and the MNA-SF showed that 49.7% of the patients had nutritional risk and 14.67% were malnourished [16]. This result was similar to the CSPEN study.

The two methods showed that patients with nutritional risk were older than patients without nutritional risk. This result also illustrates the necessity to consider age as an independent risk factor when using the NRS 2002 [17]. Many studies have reported that nutritional risk rates in departments of gastroenterology are higher than in other departments [18]. The NRS 2002 screening in this study showed similar results. Patients in the departments of gastroenterology, hematology, and pneumology had the highest nutritional risk rates. Patients in neurology, endocrinology and cardiology department had lower rates in comparison. The main diseases for patients in the department of gastroenterology included cancer, hemorrhage of the digestive tract, acute inflammation of the biliary system, gastrointestinal tract polyps, and other gastrointestinal tract diseases. Because the nature of the diseases affected appetite and the ability to digest and absorb nutrients, weight-loss, reduced feeding, and severe wasting was more common. The nutritional damage rating in this department was higher than in other departments. Further, the department of gastroenterology had a higher incidence of acute disease, which would logically increase the acuteness level rating. Patients in the department of pneumology were commonly admitted due to acute lung infections. Elderly people were at a high risk for acute pneumonia, severe hypoxemia, and stress states driving negative oxygen balance, which significantly affected nutritional status. Hematological malignancy was common in patients of the department of hematology, and the acuteness of this disease increased the rating in the NRS 2002.

Nutritional support was an important component of clinical nutritional treatment. After the 1990s, there were clear developments in the study of clinical nutritional support, and the inappropriate use of clinical nutritional support was taken seriously. Many studies into the application of nutritional support showed differences in the inappropriate use of nutritional support between different departments and hospitals [19]. In this study, the overall nutritional support rate for the 746 patients was 16.49%, and the nutritional support rate for patients with nutritional risk by NRS 2002 and MNA-SF was 29.63% and 28.57%, respectively. Compared to the 40% nutritional risk rate, the application of nutritional support was clearly insufficient. In the two methods, the nutritional support rate for patients without nutritional risk was 7.8%(35/449) and 6.96%(29/417). The data showed that patients with normal nutritional status were still receiving nutritional support. Through analysis of nutritional support, this study revealed that overall, PN was the main method. Only a few patients received EN as the main method of nutritional support. In addition, for patients in need, doctors did not utilize sufficient nutritional support. Due to research, there could be certain reasons: firstly, clinical doctors had an insufficient understanding of nutritional support; secondly, patients were unaware of the importance of nutritional support and rejected the doctors’ advices; additionally, the cost of nutritional support drugs and the health care reimbursement policy limited the prevalence of nutritional support. More questionnaires and clinical studies are needed to prove this hypothesis in the future. From the results of our research, for patients at risk of nutrition, timely nutrition screening and assessment and appropriate nutrition interventions were important and need to be applied in a better way. For the patients with normal nutritional status, hospitals should raise the standard for nutritional support to prevent over-application of nutritional support. Although PN was the main choice for nutritional support in this study, many studies have shown that for patients without contraindication to EN who were in need of nutritional support in terms of clinical results or cost-benefit analysis, EN was preferable to PN [20–22]. Therefore, doctors should deepen their knowledge of clinical nutritional support to facilitate the choice of more reasonable support solutions for patients.

This study showed that in patients with nutritional risk, nutritional support decreased the HLOS (*P*<0.05). After excluding 11 cases whose HLOS prolonged for non-clinical reasons, for the cases with nutritional risk, patients with nutritional support stayed in the hospital shorter than the patients without nutritional support. The result was proved in NRS 2002 with MNA-SF. On the other hand, for patients with normal nutritional status, we had no further research whether inappropriate nutritional support can affect HLOS. We need to pay attention to this aspect in our future research.

This study found that the incidence of infectious complications in patients with nutritional support was lower than that of patients without nutritional support. To further analyze the effects of nutritional support on the incidence of infectious complications, we ran a multiple regression analysis. After controlling for age, gender, BMI, acuteness of disease, nutritional status rating, and HLOS, results showed that nutritional support reduced the incidence of infectious complications. This result was similar to reports from other large-sample studies [18, 23, 24]. In addition, this study revealed that in the NRS 2002, disease rates related to nutritional support in patients with nutritional risk was 5.05%, and in the MNA-SF the rate was 6.08%. Among patients with nutritional risk, the application of nutritional support did not increase the occurrence of complications.

There are some limitations to be considered in our study. First, it is a single center study. The conditions vary among regions and hospitals. The results of our study might not be representative for all hospitalized patients. In order to be able to extract reliable conclusions, a multi-center study is needed in order to limit the differences on the prevalence of nutritional risk and malnutrition, which has been found to be influenced by different countries and hospitals. Secondly, nutritional support rate is far from sufficient for the patients in our study. Most patients do not receive adequate nutritional support. The occurrence of complications is influenced by the rate of nutritional support which affects the generalizability of our results. Thirdly, laboratory tests were carried out during the first 72 hours of admission. However, a large proportion of patients did not arrange for the retaken of the laboratory tests. We could not to confirm whether nutritional support had an impact on them. We are still continuing this clinical study. In the future research, this problem will be further solved.

## Conclusion

By conducting nutritional risk screening and research on the application of nutritional support for elderly patients, this study determined the aspects that require improvement in clinical treatment regarding nutritional support. Through analysis of the relationships between nutritional risk, nutritional support, and clinical results, the study found that nutritional support can reduce HLOS and decrease the incidence of infectious complications in elderly patients with nutritional risk and malnutrition, which provides evidence for the application of nutritional support in clinical treatment.

## Supporting information

S1 Table Analysis of nutritional support. (DOCX)

S2 Table STROBE_checklist. (DOCX)
