# Supplementary material for: Screening and application of nutritional support in elderly hospitalized patients of a tertiary care hospital in China
Source: PLoS One. 2019 Mar 8;14(3):e0213076. doi: 10.1371/journal.pone.0213076 (PMC6407756; doi:10.1371/journal.pone.0213076)
Supplement: S1 Table — (DOCX) [file pone.0213076.s001.docx]

**S2 Table. Analysis of nutritional support**

|  | **Nutritional support** | | **Ways of nutritional support** | | |
| --- | --- | --- | --- | --- | --- |
|  | **Without nutritional support, n=623** | **With nutritional support**, **n=123** | **PN**, **n=82** | **EN**, **n=16** | **PN+EN**, **n=25** |
| **Age** | 76.92±6.77 | 79.19±7.67 | 78.23±7.62 | 80.56±7.43 | 81.44±7.65 |
| **Sex** |  |  |  |  |  |
| Male | 400（81.47%） | 91（18.53%） | 60（65.93%） | 10（10.99%） | 21（23.08%） |
| Female | 223（87.45%） | 32（12.55%） | 22（68.75%） | 6（18.75%） | 4（12.50%） |
| **Department** |  |  |  |  |  |
| Neurology Department | 106（86.18%） | 17（13.82%） | 9（52.94%） | 3（17.65%） | 5（29.41%） |
| Hematology Department | 48（75.00%） | 16（25.00%） | 14（87.50%） | 0（0%） | 2（12.50%） |
| Pneumology Department | 74（82.22%） | 16（17.78%） | 10（62.50%） | 3（18.75%） | 3（18.75%） |
| Gastroenterology Department | 125（71.84%） | 49（28.16%） | 33（67.35%） | 5（10.20%） | 11（22.45%） |
| Endocrinology Department | 60（90.91%） | 6（9.09%） | 6（100.00%） | 0（0%） | 0（0%） |
| Cardiology Department | 210（91.70%） | 19（8.30%） | 10（52.63%） | 5（26.32%） | 4（21.05%） |
