# Supplementary material for: Screening and application of nutritional support in elderly hospitalized patients of a tertiary care hospital in China
Source: PLoS One. 2019 Mar 8;14(3):e0213076. doi: 10.1371/journal.pone.0213076 (PMC6407756; doi:10.1371/journal.pone.0213076)
Supplement: S2 Table — (DOCX) [file pone.0213076.s002.docx]

S2 Table. STROBE_checklist

|  | Item No. | Recommendation | Page  No. | Relevant text from manuscript |  |
| --- | --- | --- | --- | --- | --- |
| **Title and abstract** | 1 | (*a*) Indicate the study’s design with a commonly used term in the title or the abstract | 1 | Screening and Application of Nutritional Support in Elderly Hospitalized Patients of a Tertiary Care Hospital in China |  |
|  |  | (*b*) Provide in the abstract an informative and balanced summary of what was done and what was found | 5 | Undernourishment and nutritional risk in elderly patients at hospital admission is a common occurrence. In the current study, the nutritional risk rate in the Gastroenterology department was higher than in other departments. Patients with normal nutritional status were still receiving nutritional support. Overall, there is a need to better apply nutritional support in the clinical treatment of elderly patients. In elderly patients with nutritional risk and malnutrition, nutritional support reduced the length of hospital stay and the incidence of infectious complications. |  |
| Introduction | | | |  |  |
| Background/rationale | 2 | Explain the scientific background and rationale for the investigation being reported | 6 | Since the practice of parenteral and enteral nutritional support was applied in clinical treatment, the application of nutritional support has experienced rapid development. The theories and methods behind nutritional support are constantly evolving. For patients who need nutritional support, choice of the appropriate method can enhance the recovery of patients and significantly improve clinical results and prognosis [1-3]. Due to a higher nutritional risk rate and functional degeneration, patients with multiple conditions are more common among elderly patients, who are more susceptible to disease and malnutrition. This makes these patients a target for nutritional support [4, 5]. Studies on the effects of nutritional support on clinical results have mainly focused on the nutritional status of patients, the tolerance of clinical treatments, rate of complications, hospital length of stay (HLOS), re-admission rate, mortality rate, and the cost of care [6, 7]. Results of the studies vary, and there is disagreement over the timing and methods for nutritional support [8, 9]. | |
| Objectives | 3 | State specific objectives, including any prespecified hypotheses | 7 | This study used the NRS 2002 and MNA-SF to conduct screening on 746 elderly hospitalized patients, evaluating the nutritional status of the patients and studying the effects of nutrition-al support on clinical results. | |
| Methods | | | |  |  |
| Study design | 4 | Present key elements of study design early in the paper | 7 | The study was a prospective observational study in the geriatric medicine department of the Qilu hospital of Shandong University. Recruitment was carried out among patients admitted consecutively to the wards from March 2012 to the end of March 2015. | |
| Setting | 5 | Describe the setting, locations, and relevant dates, including periods of recruitment, exposure, follow-up, and data collection | 7 | Recruitment was carried out among patients admitted consecutively to the wards from March 2012 to the end of March 2015. | |
| Participants | 6 | (*a*) *Cohort study*—Give the eligibility criteria, and the sources and methods of selection of participants. Describe methods of follow-up  *Case-control study*—Give the eligibility criteria, and the sources and methods of case ascertainment and control selection. Give the rationale for the choice of cases and controls  *Cross-sectional study*—Give the eligibility criteria, and the sources and methods of selection of participants | 7，8 | Inclusion criteria included: Age ≥ 65; scheduled to stay in the hospital for at least 3 days; no plan to conduct surgery after hospitalization; consent to participate in the study; must have under-gone nutritional screening within 24h after hospitalization.  Exclusion criteria included: excluded for nutritional risk screening; having surgery during hospitalization; patient with critical illness, acute disease or infection, needing treatment prior to nutritional assessment at the time of admission; HLOS < 3d; dropped out during the study; incomplete data (presented as follows). | |
|  |  | (*b*) *Cohort study*—For matched studies, give matching criteria and number of exposed and unexposed  *Case-control study*—For matched studies, give matching criteria and the number of controls per case | 9，10，14，17 | NRS 2002 and MNA-SF were used to evaluate patient nutritional status within 48h of hospitalization. NRS 2002 consists of three sections: impaired nutritional status, severity of disease and age. It contains a total of 7 points. Impaired nutritional status is scored from 0~3 according to changes of Body Mass Index (BMI), weight loss and food intake. Severity of disease is scored 0~3 ac-cording to different kinds of disease. If age ≥70 years: add 1 to the total score. Patients with a score ≥ 3 have nutritional risk and in need of nutritional support with weekly review. Patients with a score < 3 do not have nutritional risk and need no intervention. However, reviews should be conducted after one week. [10]  MNA-SF consists of six sections: appetite or eating problem, recent weight loss, mobility impairment, acute illness/ stress, dementia or depression and BMI. It contains a total of 14 total points; a score of 12-14 is within the normal range, 8-11 indicates risk of malnutrition, and ≤ 7 indicates malnutrition. [11] The standard measurement for malnutrition is a BMI of < 18.5kg/m2.  Parenteral nutrition supplies daily nutritional requirements from the peripheral vein or a central venous catheter, with non-protein calories ≥ 10kcal/kg/d for more than 3 days. Enteral nutrition includes oral nutritional supplements (ONS) as well as tube feeding via nasogastric, nasal-enteral, or percutaneous tube, with non-protein calories ≥ 10kcal/kg/d for more than 3 days.  Of the 746 patients, there were 41 cases (5.5%) of malnutrition (BMI < 18.5kg/m2) and nutritional risk was 39.61% (297/746) (using the NRS 2002 results). Using the MNA-SF to evaluate the nutritional status of patients, the study found that the ratio for patients with normal nutritional status was 55.9% (417/746), the malnutrition rate was 33.38% (249/746), the occurrence rate of malnutrition was 10.72%, and the overall nutritional risk was 44.1% (329/746) (see Fig 1).  Of the 746 cases, 123 patients (16.49%) received nutritional support which included 82 PN, 16 EN, and 25 combined application. | |
| Variables | 7 | Clearly define all outcomes, exposures, predictors, potential confounders, and effect modifiers. Give diagnostic criteria, if applicable | 10，11 | The clinical results included infectious complications, non-infectious complications, nutritional support-related complications, HLOS, and the total cost of hospitalization.  Infectious complications include localized or systemic conditions resulting from an adverse reaction to the presence of an infectious agent(s) or its toxin(s); there must be no evidence that the infection was present or incubating at the time of admission to the hospital (pneumonia, urinary tract infection, intraperitoneal infection, catheter-related infection, other infection). [13]  Non-infectious complications included new diseases and conditions after hospitalization (anemia, myocardial infarction, organ failure, etc.), caused by primary disease and weakness, excluding infection induction.  Nutritional support-related complications include gastrointestinal complications (nausea, vomiting, diarrhea, bloating, constipation), metabolic complications (hyperglycemia, hyperlipidemia, dehydration, liquid retention, hypophosphatemia, nutrition-associated liver disease, etc.), infectious complications (aspiration pneumonia, central venous catheter related infection, etc.), mechanical complications (gastric perforation, ulcer, intestinal obstruction and nutrient tube blockage, central venous catheter related injuries and embolism, etc.) caused by nutritional support. | |
| Data sources/ measurement | 8* | For each variable of interest, give sources of data and details of methods of assessment (measurement). Describe comparability of assessment methods if there is more than one group | 8，9 | Patients newly admitted were asked whether they would like to participate in this investigation. Personal information (age, sex, ethnicity, department, date of admission, primary diagnosis, co-existing comorbidities) were collected. After their agreement, nutritional screening was performed by the same researchers after standard training. Patients’ weight and height from admission to discharge were measured also by the researchers with the same standard scale: before meals in the morning, with shoes off and wearing a hospital gown. The height of the patient was measured to the nearest 0.5 cm, and body weight to the nearest 0.5kg. The following laboratory tests were carried out using standard methods within 72h after hospitalization: hemoglobin (Hb), total lymphocyte count (TLC), and albumin (Alb). The patients’ nutritional supports being delivered and clinical results were also collected from their medical records. The data-collection should be per-formed until the time of patients’ discharge. Data were evaluated independently by two individuals before entering the information into the electronic database. | |
| Bias | 9 | Describe any efforts to address potential sources of bias | 8，9 | After their agreement, nutritional screening was performed by the same researchers after standard training. Patients’ weight and height from admission to discharge were measured also by the researchers with the same standard scale: before meals in the morning, with shoes off and wearing a hospital gown. The height of the patient was measured to the nearest 0.5 cm, and body weight to the nearest 0.5kg. The following laboratory tests were carried out using standard methods within 72h after hospitalization: hemoglobin (Hb), total lymphocyte count (TLC), and albumin (Alb). The patients’ nutritional supports being delivered and clinical results were also collected from their medical records. The data-collection should be per-formed until the time of patients’ discharge. Data were evaluated independently by two individuals before entering the information into the electronic database. | |
| Study size | 10 | Explain how the study size was arrived at |  | N/A | |
| Quantitative variables | 11 | Explain how quantitative variables were handled in the analyses. If applicable, describe which groupings were chosen and why | 12 | Quantitative data were expressed as mean ± standard deviation, and qualitative data were expressed as proportions. Differences in Quantitative data were analyzed using analysis of variance. |  |
| Statistical methods | 12 | (*a*) Describe all statistical methods, including those used to control for confounding | 12 | Statistical analyses were performed with SPSS 20.0 (SPSS Inc., Chicago, IL, USA). Quantitative data were expressed as mean ± standard deviation, and qualitative data were expressed as proportions. Differences in Quantitative data were analyzed using analysis of variance. Differences in qualitative data were measured by chi-square test or Fisher’s exact test. Multivariate statistical analysis of the factors affecting clinical outcomes were measured by Logistic multiple regression analysis. P < 0.05 was considered statistically significant. |  |
|  |  | (*b*) Describe any methods used to examine subgroups and interactions |  | N/A |  |
|  |  | (*c*) Explain how missing data were addressed | 7，8 | Exclusion criteria included: excluded for nutritional risk screening; having surgery during hospitalization; patient with critical illness, acute disease or infection, needing treatment prior to nutritional assessment at the time of admission; HLOS < 3d; dropped out during the study; incomplete data (presented as follows). |  |
|  |  | (*d*) *Cohort study*—If applicable, explain how loss to follow-up was addressed  *Case-control study*—If applicable, explain how matching of cases and controls was addressed  *Cross-sectional study*—If applicable, describe analytical methods taking account of sampling strategy | 7，8 | Exclusion criteria included: excluded for nutritional risk screening; having surgery during hospitalization; patient with critical illness, acute disease or infection, needing treatment prior to nutritional assessment at the time of admission; HLOS < 3d; dropped out during the study; incomplete data (presented as follows). |  |
|  |  | (*e*) Describe any sensitivity analyses |  | N/A |  |
| Results | | | | |  |
| Participants | 13* | (a) Report numbers of individuals at each stage of study—eg numbers potentially eligible, examined for eligibility, confirmed eligible, included in the study, completing follow-up, and analysed | 13 | There were 746 elderly patients included in this study with an average age of 77.29 ± 6.97 and BMI of 23.67 ± 3.49 kg/m^2^. |  |
|  |  | (b) Give reasons for non-participation at each stage |  | N/A |  |
|  |  | (c) Consider use of a flow diagram |  | N/A |  |
| Descriptive data | 14* | (a) Give characteristics of study participants (eg demographic, clinical, social) and information on exposures and potential confounders | 13 | There were 746 elderly patients included in this study with an average age of 77.29 ± 6.97 and BMI of 23.67 ± 3.49 kg/m2. The sample included 491 male patients with an average age of 77.62 ± 7.12 and a BMI of 23.87 ± 3.36 kg/m2, and 225 female patients with an average age of 76.66 ± 6.66 and a BMI of 23.67 ± 3.73 kg/m2 (see Table 1). |  |
|  |  | (b) Indicate number of participants with missing data for each variable of interest |  | N/A |  |
|  |  | (c) *Cohort study*—Summarise follow-up time (eg, average and total amount) | 7，13 | Recruitment was carried out among patients admitted consecutively to the wards from March 2012 to the end of March 2015.  There were 746 elderly patients included in this study with an average age of 77.29 ± 6.97 and BMI of 23.67 ± 3.49 kg/m2. |  |
| Outcome data | 15* | Cohort study—Report numbers of outcome events or summary measures over time | 18，19，20 | Based on the NRS 2002 results, of the 297 cases with nutritional risk, 11 cases were excluded due to a HLOS that was pro-longed for non-clinical reasons. For the remaining 286 cases, patients with nutritional support stayed in the hospital for an average of 12.22 ± 5.48 days, which was shorter than the patients without nutritional support (14.35 ± 6.19 days, t = -2.839, P<0.05). MNA-SF screening in patients with nutritional risk (after excluding the 11 cases) revealed that the HLOS of patients with nutritional support was (11.94 ± 5.18 days, which was shorter than patients without nutritional support (14.41 ± 6.44 days, t = -3.529, P<0.05).  At NRS 2002 screening, of the 297 patients with nutritional risk, complications occurred in 56 cases (18.86%), the incidence of infectious complications was 14.81% (44/297), the incidence of non-infectious complications was 1.68% (5/297), and the incidence of complications related to nutritional support was 5.05% (15/297).  At MNA-SF screening, of the 329 patients with nutritional risk, the overall incidence of complications was 20.36% (67/329), in which the incidence of infectious complications was 17.33% (57/329) and the incidence of non-infectious complications was 2.74% (9/329). The nutritional support group had a lower incidence of infectious complications than the non-nutritional support group (P<0.05). |  |
|  |  | *Case-control study—*Report numbers in each exposure category, or summary measures of exposure |  | N/A |  |
|  |  | *Cross-sectional study—*Report numbers of outcome events or summary measures |  | N/A |  |
| Main results | 16 | (*a*) Give unadjusted estimates and, if applicable, confounder-adjusted estimates and their precision (eg, 95% confidence interval). Make clear which confounders were adjusted for and why they were included | 18-21 | Based on the NRS 2002 results, of the 297 cases with nutritional risk, 11 cases were excluded due to a HLOS that was pro-longed for non-clinical reasons. For the remaining 286 cases, patients with nutritional support stayed in the hospital for an average of 12.22 ± 5.48 days, which was shorter than the patients without nutritional support (14.35 ± 6.19 days, t = -2.839, P<0.05). MNA-SF screening in patients with nutritional risk (after excluding the 11 cases) revealed that the HLOS of patients with nutritional support was (11.94 ± 5.18 days, which was shorter than patients without nutritional support (14.41 ± 6.44 days, t = -3.529, P<0.05).  The nutritional support group had a lower incidence of infectious complications than the non-nutritional support group, (P < 0.05). Regarding the incidence of non-infectious complications and overall complications, the difference between the two groups was not statistically significant (P > 0.05).  The nutritional support group had a lower incidence of infectious complications than the non-nutritional support group (P<0.05). Details regarding complications in the nutritional support group and the non-nutritional support group (MNA-SF) are shown in Table 5.  we ran a multivariate regression analysis on the factors that affect infectious complications (age, sex, BMI, nutritional support status, rating of illness degree, nutritional status rating, and HLOS). The results showed that the rating for illness, nutritional status rating, and HLOS were significantly related to the incidence of complications (P < 0.05, Table 6). |  |
|  |  | (*b*) Report category boundaries when continuous variables were categorized |  | N/A |  |
|  |  | (*c*) If relevant, consider translating estimates of relative risk into absolute risk for a meaningful time period |  | N/A |  |
| Other analyses | 17 | Report other analyses done—eg analyses of subgroups and interactions, and sensitivity analyses |  | N/A |  |
| Discussion | | | | |  |
| Key results | 18 | Summarise key results with reference to study objectives | 22 | Our results suggest that undernourishment and nutritional risk in elderly at hospital admission is a common occurrence. Patients with nutritional risk are more likely to have a longer hospital stay. Nutritional support decreases the length of hospital stay and reduces the number of patients with infectious complications. |  |
| Limitations | 19 | Discuss limitations of the study, taking into account sources of potential bias or imprecision. Discuss both direction and magnitude of any potential bias | 27，28 | There are some limitations to be considered in our study. First, it is a single center study. The conditions vary among regions and hospitals. The results of our study might not be representative for all hospitalized patients. In order to be able to extract reliable conclusions, a multi-center study is needed in order to limit the differences on the prevalence of nutritional risk and malnutrition, which has been found to be influenced by different countries and hospitals. Secondly, nutritional support rate is far from sufficient for the patients in our study. Most patients do not receive adequate nutritional support. The occurrence of complications is influenced by the rate of nutritional support which affects the generalizability of our results. Thirdly, laboratory tests were carried out during the first 72 hours of admission. However, a large proportion of patients did not arrange for the retaken of the laboratory tests. We could not to confirm whether nutritional support had an impact on them. We are still continuing this clinical study. In the future re-search, this problem will be further solved. |  |
| Interpretation | 20 | Give a cautious overall interpretation of results considering objectives, limitations, multiplicity of analyses, results from similar studies, and other relevant evidence | 26 | By conducting nutritional risk screening and research on the application of nutritional support for elderly patients, this study determined the aspects that require improvement in clinical treatment regarding nutritional support. Through analysis of the relationships between nutritional risk, nutritional support, and clinical results, the study found that nutritional support can reduce HLOS and decrease the incidence of infectious complications, which provides evidence for the application of nutritional support in clinical treatment. |  |
| Generalisability | 21 | Discuss the generalisability (external validity) of the study results | 28，29 | Through analysis of the relationships between nutritional risk, nutritional support, and clinical results, the study found that nutritional support can reduce HLOS and decrease the incidence of infectious complications in elderly patients with nutritional risk and malnutrition, which provides evidence for the application of nutritional support in clinical treatment. |  |
| Other information | |  | | |  |
| Funding | 22 | Give the source of funding and the role of the funders for the present study and, if applicable, for the original study on which the present article is based | 29 | We would like to thank the participants, without whom this study would not have been possible. We also thank our colleges in Yeelo Research of Qilu Hospital of Shandong University for providing helpful support and advice on the research. |  |

*Give information separately for cases and controls in case-control studies and, if applicable, for exposed and unexposed groups in cohort and cross-sectional studies.

**Note:** An Explanation and Elaboration article discusses each checklist item and gives methodological background and published examples of transparent reporting. The STROBE checklist is best used in conjunction with this article (freely available on the Web sites of PLoS Medicine at http://www.plosmedicine.org/, Annals of Internal Medicine at http://www.annals.org/, and Epidemiology at http://www.epidem.com/). Information on the STROBE Initiative is available at www.strobe-statement.org.
